# A Review of Magnetic Elastomers and Their Role in Soft Robotics

**DOI:** 10.3389/frobt.2020.588391

**Published:** 2020-10-23

**Authors:** Nicholas Bira, Pallavi Dhagat, Joseph R. Davidson

**Affiliations:** ^1^Collaborative Robotics and Intelligent Systems Institute, Oregon State University, Corvallis, OR, United States; ^2^School of Electrical Engineering and Computer Science, Oregon State University, Corvallis, OR, United States

**Keywords:** magnetic elastomers (ME), magnetorheological elastomer (MRE), magnetic powder, elastomers, magnetism and electricity, soft robotics, magnetic composite

## Abstract

Soft robotics as a field of study incorporates different mechanisms, control schemes, as well as multifunctional materials to realize robots able to perform tasks inaccessible to traditional rigid robots. Conventional methods for controlling soft robots include pneumatic or hydraulic pressure sources, and some more recent methods involve temperature and voltage control to enact shape change. Magnetism was more recently introduced as a building block for soft robotic design and control, with recent publications incorporating magnetorheological fluids and magnetic particles in elastomers, to realize some of the same objectives present in more traditional soft robotics research. This review attempts to organize and emphasize the existing work with magnetism and soft robotics, specifically studies on magnetic elastomers, while highlighting potential avenues for further research enabled by these advances.

## 1. Introduction

The field of soft robotics contains many ongoing investigations concerning the design, modeling, manufacturing, and control of soft bodies. There have been years of research involving the use of pressure differentials to enable shape change, but there is no commonly agreed upon solution to such soft robotic design challenges as autonomy, robustness, and portability. There is a continuous demand for higher actuation strengths, more freedom of movement, less power consumption, and greater durability in the design of these soft robots (Lu and Kim, 2014; Whitesides, 2018). Research into these subjects continues to expand, and alternative mechanisms, materials, and strategies are actively developing to enable fully soft and functional robots.

A challenging area for soft robotics is reducing robotic tethering, or the reliance of a soft robot on a base station for power, pressure, and other forms of regulation (Rus and Tolley, 2015; Whitesides, 2018). Review articles characterize soft robotic actuation types as tendon-driven, fluidically actuated, or using electro-active polymers (EAP) (Lee et al., 2017). EAPs are well-established in soft robotic systems, enabling electrical control of the material properties and shape of elastomers. Remote actuation, power delivery, and control could help overcome the current limitation of most soft robotic systems' reliance upon a tether to a base station, and would likely fall outside of these traditional classifications. Remote inputs, or on-board control methods, could build on existing control methodologies, which often utilize standard, hard electromagnetic motors to actuate soft robots (Rus and Tolley, 2018).

One area of soft robotics research currently seeing rapid innovation involves the use of magnetism and magnetic materials to realize soft robotic goals. Compared to some of the actuation methods previously discussed (i.e., tendon-driven, fluid systems, electro-active materials), relatively few publications exist that demonstrate the use of magnetism to remotely modify the mechanical properties of an elastomer in a soft robotic system; those that do demonstrate many desirable traits, such as remote actuation for tetherless and controlled-deformation soft robots (Kim et al., 2011; Hu et al., 2018; Zhang and Diller, 2018; Joyee and Pan, 2019).

This paper is concerned primarily with magnetic elastomers (MEs), elastic, magnetic smart materials designed to respond to magnetic fields which affect their geometry, as well as their rheology and electromagnetic properties. First, we discuss the historical use of MEs in research, including previous applications and directions, before focusing on contemporary uses of MEs in soft robotic systems. We sort different types of MEs, differentiating by both application as well as materials used, and discuss future directions and opportunities for MEs in soft robotics. Overall, this review seeks to summarize and discuss the current state of work regarding MEs and their role in soft robotics, as well as identify promising research directions using MEs.

## 2. Review

**Figure 1:**
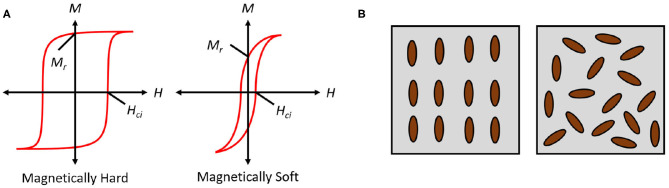
**(A)** The magnetization, *M*, vs. the applied field, *H*, for a hard (left) and soft (right) magnetic material. On these plots, *M*_*r*_ represents the remanence magnetization (i.e., magnetization in the absence of a field). *H*_*ci*_ represents coercivity. **(B)** A representation of elastomers cured with (left) and without (right) an applied field during curing. Application of a field yields an anisotropic ME with particles aligned in the direction of the applied field. In the absence of a field, an isotropic ME, with randomly oriented particles, is obtained.

This section reviews past and current research on magnetic elastomers and their applications in soft robotics. It is structured into subsections based on material properties and characterization of MEs, elastomers made from hard magnetic particles (hMEs), as well as elastomers made from soft magnetic particles (sMEs) and their applications in soft robotics. The terms soft and hard magnetism relate to the intrinsic coercivity (*H*_*ci*_) of these particles, i.e., the external applied field necessary to demagnetize the material (see Figure 1A). Hard magnets require a larger applied field to demagnetize and retain their magnetization in the absence of a field. Soft magnetic materials, in contrast, are easier to demagnetize and have a low remanence magnetization.

Magnetic elastomers in the literature are referred to by a variety of terms, usually depending on the application. Some of these terms include magnetorheological elastomers (MREs), magnetically responsive elastomers, shape-programmable magnetic matter (Lum et al., 2016), or magnetoactive elastomers (MAEs) (Bowen et al., 2015). In some cases, it may be appropriate to refer to the material as an MRE when the focus or study explicitly concerns the material properties of the ME and how to control them. In other cases, the term magneto-morphological elastomers (MMEs) may be more appropriate, as many studies concerning soft robotics typically focus on shape deformation, over material property variation. For simplicity, we refer to all of these materials as magnetic elastomers (MEs) with differentiation given for whether they utilize magnetically soft or magnetically hard particles (sMEs or hMEs, respectively).

### 2.1. Materials Characterization

MEs typically consist of an elastomer with embedded hard or soft magnetic particles, occasionally with other additives to achieve different performance goals (Davis, 1999; Lokander and Stenberg, 2003; Gong et al., 2005; Kallio, 2005; Chen et al., 2007; Zajac et al., 2010). The elastomer, usually silicone or polyurethane rubber, is mixed with a certain volume percentage of the additives and magnetic particles. The resulting mixture is then cured, usually in a heat chamber, to prevent the particles from settling out of the mixture. Other methods for producing MEs from flexible materials involve ultraviolet curing and 3D printing, such as in masked stereolithography (MSLA) 3D printing (Hellebrekers et al., 2020). MEs can be cured under the influence of an external magnetic field, which aligns the magnetic particles within into chains (Jolly et al., 1996a,b; Carlson and Jolly, 2000; Bellan and Bossis, 2002). These types of MEs are referred to as anisotropic, while isotropic MEs are cured without an external field, and have random orientation of the particles within (Davis, 1999) (see Figure 1B). In anisotropic samples, the MEs demonstrate enhanced properties, improving their responsiveness to external applied fields in the form of greater shear stresses and larger magnetic attraction forces (Böse, 2007; Böse and Röder, 2009; Hadzir et al., 2019). Alignment of the particles under an applied field is also dependent on the viscosity of the elastomer during curing, and some research models this interaction to better predict alignment success and the resulting magnetic properties (Ciambella et al., 2017). Some researchers attempted to model and predict the magnetic and physical properties of isotropic MEs (Dorfmann and Ogden, 2003), having some success with modeling the shear stiffness of a cylindrical ME. When subjected to a sufficiently strong external field, it is possible for an isotropic ME to become anisotropic, as the particles break their orientation and rotate within the elastomer matrix to become aligned (Coquelle and Bossis, 2006).

These properties of MEs, specifically their microstructure, have been confirmed through various methods, such as scanning electron microscopy, which can show the isotropic or anisotropic arrangement (Gong et al., 2005; Nayak et al., 2015; Jung et al., 2016; Damiani and Sun, 2017). Energy-dispersive X-ray spectroscopy (Nayak et al., 2015; Jung et al., 2016) and three-dimensional nano-computed tomography imaging have also been used to validate the alignment of the magnetic particles within the matrix (Damiani and Sun, 2017).

### 2.2. MEs in Damping Systems and Material Property Control

A subset of MEs, referred to extensively in the literature as MREs, are a class of materials able to change both their shape and material properties when exposed to a magnetic field (Li et al., 2014). Research surrounding MREs exists primarily within the areas of vibration damping and control. The magnetostrictive (change in shape during the application of a magnetic field) properties of MREs are of interest for application in damping systems and for precision control of vibration mitigation (Zhou, 2003). Other research in the area of MREs and damping control include: inserting carbon nanotubes and investigating their effect on the MRE's shear modulus (Zhao et al., 2019a,b), adding carbon black to enhance desirable damping properties (Nayak et al., 2015), studying the impact of magnetic anisotropy on storage modulus (Jung et al., 2016), studying the impact of acetone on particle alignment and storage modulus (Damiani and Sun, 2017), investigating the contributions of different additives, such as ammonium bicarbonate on material properties (Ju et al., 2012), inspecting the impact of heat and radiation on MRE performance (Zhang et al., 2009b; Wan et al., 2018), exploring the role of particle volume percentage on vibration isolation (Leng et al., 2018), testing a variable stiffness MRE spring for use in a prosthetic device (Gudmundsson, 2011), and creating variable stiffness and damping isolators with MREs (Behrooz et al., 2014).

Materials known as magnetorheological fluids (MRFs) perform similar functions, but exist as a fluid instead of an elastic solid in a demagnetized state. MRFs were considered for automotive systems involving shock absorption and differential clutches (Gordaninejad and Breese, 1999; Kavlicoglu et al., 2006), and were suggested to be suitable in areas, such as earthquake mitigation in structures and car seat vibration mitigation (Jolly et al., 1999). MRFs have demonstrated uses in soft robotic systems, such as in a recent publication involving encapsulated MRF and applied magnetic fields to generate a crawling gait in an inchworm robot (Hua et al., 2020). These materials utilize the presence of a electromagnetic field to enact shape or property changes, and have various applications within soft robotics as a result. Many of the concepts explored within soft robotics utilizing MRFs could be expanded upon with MEs, since they utilize similar principles to enact rheological changes.

### 2.3. hMEs and Soft Robotic Applications

**Figure 2:**
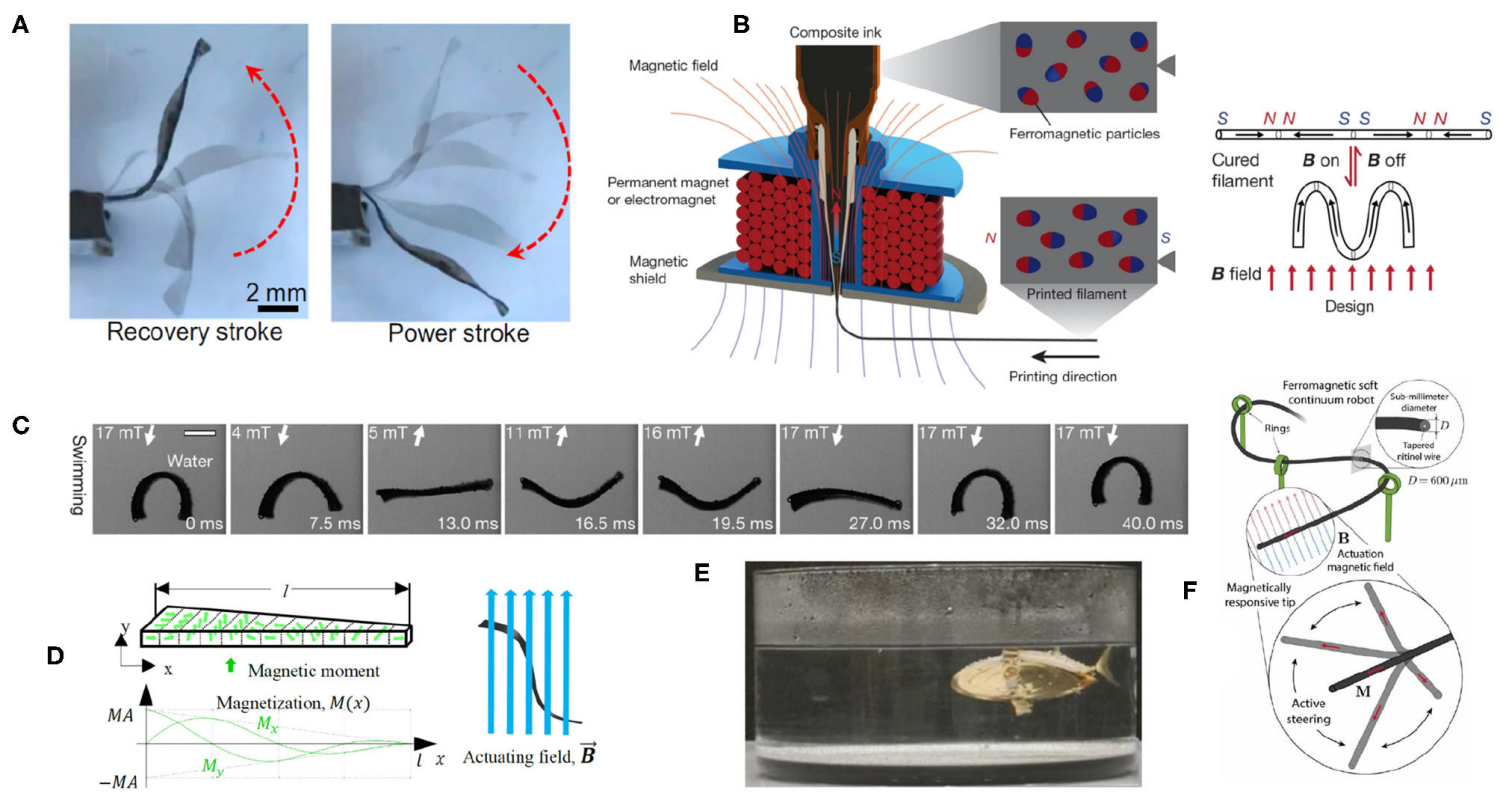
A compilation of hMEs and their soft robotic applications: **(A)** a bending hME actuator, controlled by the application of a magnetic field (Lum et al., 2016), **(B)** 3D printer head with electromagnet around the nozzle to program magnetic domains of the extruded hME throughout the print (Kim et al., 2018), **(C)** soft swimmer with varying applied fields to enable shape change and a swimming gait (Hu et al., 2018), **(D)** a triangular tail soft swimmer with varying magnetic domains to enable an undulating swimming motion (Manamanchaiyaporn et al., 2020), **(E)** origami-inspired folded fish robot swimming utilizing remote magnetic guidance (Sung et al., 2017), and **(F)** steerable hME-based wire, guided remotely (Kim et al., 2019).

The fields of bio-medicine, bio-mimicry, and robotic grasping are all targets for soft robotic research using soft actuators (Gorissen et al., 2017). Within this space, there are several promising applications for MEs, specifically hMEs, to achieve manipulation and guidance of soft actuators. There has been an increase in the number of publications concerning the use of hMEs during the past few years. All of the publications involving hard magnetic particles found by the authors utilize a specific hard magnetic powder embedded in various elastomers to achieve shape deformation, namely, NdFeB. This magnetic particle demonstrates high coercivity and remanence, and MEs with NdFeB can be fabricated with domains or regions of different magnetic alignment, allowing them to change shape and orientation when subjected to a varying (both in time or space) applied magnetic field (Lum et al., 2016) (see Figure 2A). These traits are utilized by research involving 3D printing of hMEs with embedded electromagnets around the printing nozzle to create anisotropic hMEs with variable magnetic domains within the soft robot (Kim et al., 2018). The control of magnetic alignment is achieved by curing the elastomer in the presence of a magnetic field (of appropriate strength) and switching its orientation throughout the printing process to program varying domains within a single print. Specific deformations of this fabricated hME can then be achieved upon application of a magnetic field to realize complex remote manipulation and repeatable movements, such as rolling, twisting, and folding (see Figure 2B).

Other research using similar approaches created a soft robotic swimmer composed of hMEs with varying magnetic domains. Two research groups produced millimeter-scale soft robots able to rotate, swim and roll by varying the intensity and direction of the applied magnetic field (Hu et al., 2018; Zhang and Diller, 2018) (see Figure 2C). Similar research expanded upon this approach, adding a triangular tail and undulating gait behavior (Manamanchaiyaporn et al., 2020) (see Figure 2D). In biomedical applications, a long, thin soft microbot containing hME was guided remotely within a 3D phantom vascular network (Jeon et al., 2019). A comparable work created a steerable soft robotic wire, enabling remote guidance of this wire through tortuous and narrow paths, potentially delivering optical fibers or micro-surgical tools to locations deep within a complex network of small passages (Kim et al., 2019) (see Figure 2F). This opens the possibility of varied medical uses, where magnetic fields, such as those generated by magnetic resonance imaging machines, may be used to remotely manipulate a soft robot. Another approach created a wearable, magnetic skin composed of hME for multiple applications, including eye-tracking and remote gesture control when used in coordination with other sensors (Almansouri et al., 2019). Remote inputs, or on-board control methods, could build on the existing control methodologies frequently utilizing standard electromagnetic motors to actuate origami soft robots (Rus and Tolley, 2018). Some folded, origami-based soft robots were able to achieve swimming locomotion by embedding a solid NdFeB magnet into a folded origami shape made from paper, resembling a fish (Sung et al., 2017) (see Figure 2E). While not elastic, the remote control aspect of a hard magnetic element embedded within a soft robot is a consistent approach. Remote manipulation of a folding origami structure was achieved by adhering permanent bar magnets to a pre-bent polypropylene sheet and applying a magnetic field (Bowen et al., 2015), which the authors suggest could be applied to hMEs embedded in flexible silicone origami shapes.

Soft robotic control techniques often rely on traditional mechanisms and techniques, utilizing bulky off-board solenoids, direct connections, tethers and more. Robotic motion control utilizing magnetic materials is an area of emerging interest for applications where such bulk is impractical, for example, in guided robotic surgery or robotic exploration in confined spaces. Research is ongoing to model and analytically predict elastic deformation resulting from hME materials under applied magnetic fields (Zhao et al., 2019a,b). By developing robust models for these kinds of situations, further hME-based soft actuators and remote actuation methods can be developed for different needs and applications.

### 2.4. sMEs and Soft Robotic Applications

**Figure 3:**
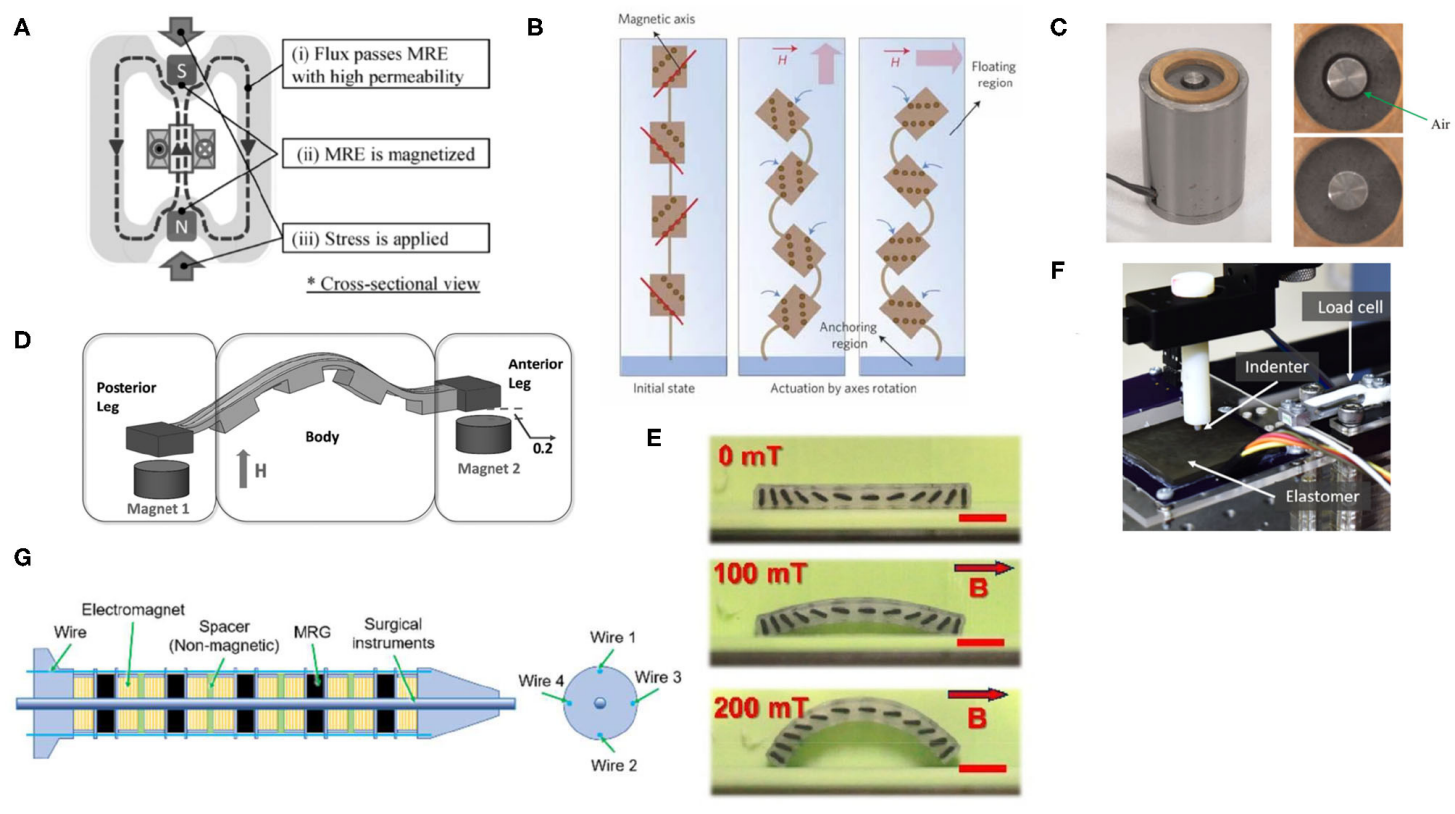
A compilation of sMEs and their soft robotic applications: **(A)** a magnetostriction-based actuator utilizing an electromagnet core to contract the sME shell (Kashima et al., 2012), **(B)** a linkage of sMEs with aligned particles at 90° offsets, enabling linkage rotation depending on the direction of the applied *H* field (Kim et al., 2011), **(C)** a pneumatic valve composed of an sME around a solid core (Böse et al., 2012), **(D)** an inchworm soft robot with sME anchor points for an inchworm-like gait (Joyee and Pan, 2019), **(E)** an applied field causing shape deformation of embedded 3D-printed magnetic elements in silicone (Qi et al., 2020), **(F)** a sME-based sensing skin coupled with Hall effect sensors and a neural network to localize deformations (Hellebrekers et al., 2020), and **(G)** a variable stiffness manipulator utilizing electromagnets and sMEs to control flexion joint angles (Kitano et al., 2020).

While some soft robotic researchers utilize the high magnetic remanence of hard magnetic materials, other work is focused on soft magnetic powders. A common material is Fe 3O 4 (iron oxide, also known as magnetite), which is relatively inexpensive and well-characterized for its soft magnetic properties. Some initial work developed an sME created from Fe 3O 4 and sought to control its elastic modulus and shear properties with an applied magnetic field (Zhou, 2003). These material traits are of significant interest to soft robotics, as selectively controlling the elastic modulus of a soft material can be crucial to the overall performance and function of a soft robot. A recent publication reported on a multi-jointed laparoscopic manipulator, utilizing an sME and electromagnets to control individual joint material properties and flexion angles (Kitano et al., 2020) (see Figure 3G).

Other researchers sought to create an actuator utilizing magnetostriction and the elastic properties of an sME, i.e., contraction of the sME when it is magnetized by an electromagnet at the center of the device (Kashima et al., 2012) (see Figure 3A). Beyond demonstrating that magnetostriction is possible with sMEs, this work demonstrates that sMEs are suitable for facilitating magnetic flux to flow through them when coupled with a source, and have the potential for inclusion in magnetic circuits. Other work created micro-actuators, making use of particle alignment to reversibly deform small bending actuators in the presence of an externally applied field (Kim et al., 2011) (see Figure 3B).

Small, swimming soft robots were made with sME heads and helical flagella, which responded to externally applied magnetic fields to enable rotation of the flagella and linear swimming (Zhang et al., 2009a). Other work created remotely movable micro-grippers made from sME and a thermally responsive material (Pacchierotti et al., 2017). These grippers were positioned using external fields, and then actuated to grasp by changing the local temperature. Similarly, Tang et al. (2018) used the heating effect of an alternating magnetic field on Fe 3O 4 to curl and grasp with a hydrogel-based sME. For terrestrial locomotion, researchers developed soft inchworm-inspired robots, capable of demonstrating an inchworm-like gait. These robots were 3D printed using MSLA techniques, and an sME was embedded within the end effectors of the soft robot to enable remote manipulation and flexion of the inchworm robot (Joyee and Pan, 2019) (see Figure 3D). Another inchworm-inspired soft robot utilized a carbonyl iron-based 3D printing filament printed into small segments of rigid, magnetically aligned regions, which were then cast in silicone to create a type of sME with distinct separation between the magnetic elements and the elastic elements (Qi et al., 2020) (see Figure 3E).

Researchers attempted to characterize the responsiveness of an sME to an applied magnetic field, producing an sME-based valve for controlling the flow of air (Böse et al., 2012) (see Figure 3C). The resulting valve was mostly rigid, with both isotropic and anisotropic sMEs providing varying valve performances. This work demonstrated that sMEs could be used in valving systems for air, as well as for liquid valving. More recently, others sought to produce a peristaltic pump, utilizing the action of multiple electromagnets compressing a tube of MRE to squeeze fluid forward (Wu et al., 2019). The same researchers also provided a numerical analysis and framework of the performance of their designed device, attempting to model and optimize its performance (Wu et al., 2020). Control of fluid flow was previously only realized in micro-fluidic systems, but with a similar concept of deforming an ME locally to displace fluid (Hilber, 2016).

In a recent publication, researchers developed a soft skin comprised of sME to sense touch and local deformations (Hellebrekers et al., 2020) (see Figure 3F). This skin utilized an array of Hall effect sensors below the skin to detect changes in the sME as it was prodded and deformed across the surface. Based on those changes, they used a neural network to interpret the output data and reproduce the state of the contacts on the sME-based skin. Alfadhel and Kosel (2015) attempted to create a cilia-inspired sensing composite material using silicone and magnetized iron nanowires for sensing applications.

## 3. Future Directions

The prior work discussed in this paper highlight a variety of significant research into the nature of MEs and their applications. More recently, MEs have begun to make their way into soft robotics publications, demonstrating unique solutions to broader soft robotic issues. Utilizing hyperelastic materials as the basis for soft magnetic hybrid materials enables unique applications within soft robotics and related fields. Molding MEs into deformable shapes through casting or 3D printing enables novel deformation and actuation driven by magnetic fields. Embedding MEs into soft robotic systems draws closer to realizing fully soft, flexible, and tetherless robotic systems with diverse applications. A summary of the various works described in this paper can be found below in Table 1.

**Table 1:** MEs in soft robotic applications.

**Application**	**Magnetic material**	**References**
sME actuator	Carbonyl iron	Kashima et al., 2012
Rheology control	Carbonyl iron	Jung et al., 2016
Micro-wire steering	NdFeB	Jeon et al., 2019
Micro-wire steering	NdFeB	Kim et al., 2019
Rotation of flagella	Cr/Ni/Au trilayer	Zhang et al., 2009a
Controllable VSDI	Carbonyl iron	Behrooz et al., 2014
Actuator positioning	Fe2O3	Pacchierotti et al., 2017
Origami folding	Bar magnet, NdFeB	Bowen et al., 2015
Origami fish steering	Bar magnet, NdFeB	Sung et al., 2017
Inchworm control	Black iron oxide	Joyee and Pan, 2019
Inchworm control	Carbonyl iron	Qi et al., 2020
Inchworm soft robot	MRF (iron)	Hua et al., 2020
sME air valve	Carbonyl iron	Böse et al., 2012
sME flexing joint	Carbonyl iron	Kitano et al., 2020
3D deformable structures	NdFeB	Kim et al., 2018
Elastic beam deformation	NdFeB	Lum et al., 2016
Millimeter unthethered swimmers	NdFeB	Zhang and Diller, 2018
Millimeter unthethered swimmers	NdFeB	Hu et al., 2018
Millimeter unthethered swimmers	NdFeB	Manamanchaiyaporn et al., 2020
Variable length linkages	Superparamagnetic Fe3O4	Kim et al., 2011
Magnetothermal shape change	Fe3O4	Tang et al., 2018
Sensing tactile skin	Nd-Pr-Fe-B	Hellebrekers et al., 2020
Sensing tactile skin	Iron nanowires	Alfadhel and Kosel, 2015
Sensing tactile skin	Iron nanowires	Alfadhel and Kosel, 2015
Wearable hME skin	NdFeB	Almansouri et al., 2019
Peristaltic pump	Carbonyl iron	Wu et al., 2019

### 3.1. Research Directions, Paths Forward

**Figure 4:**
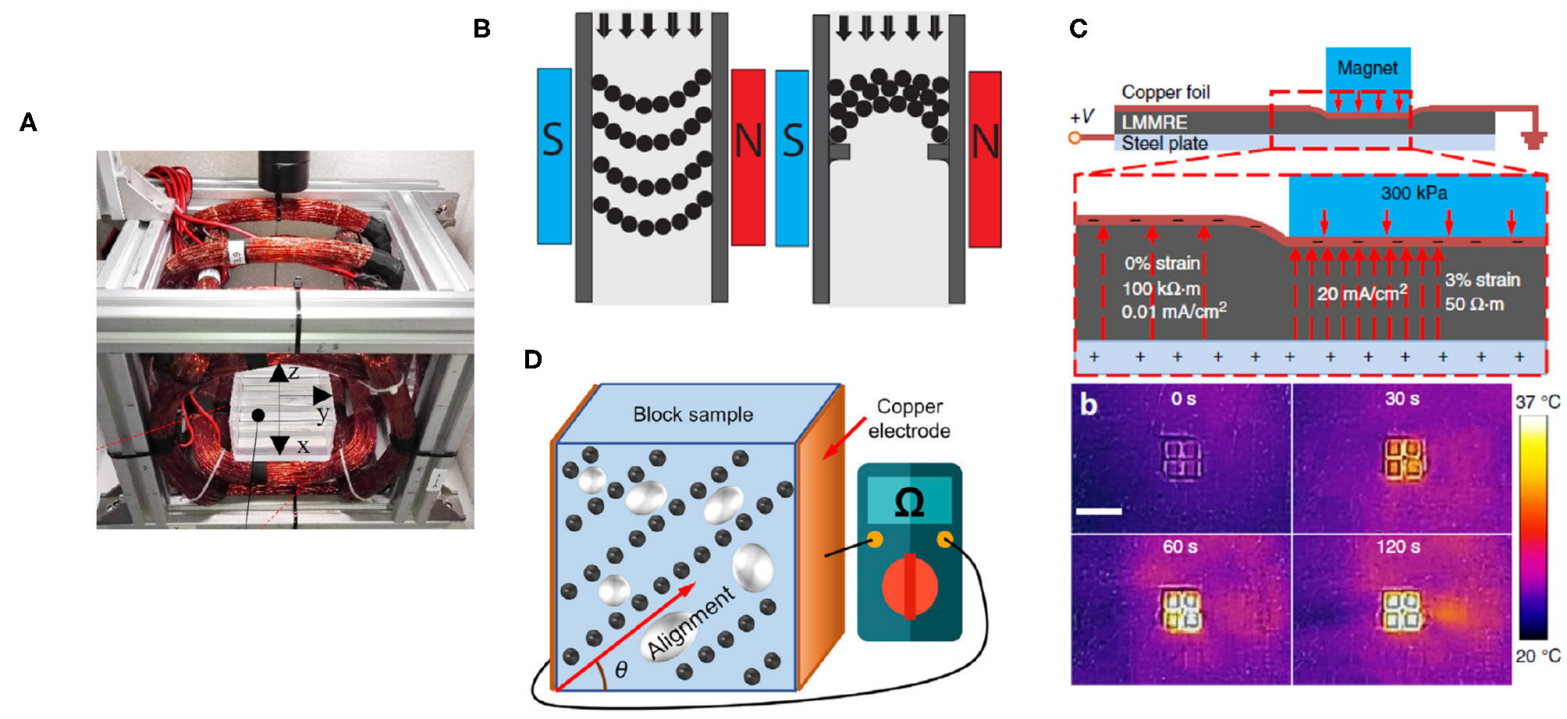
**(A)** Large, bulky magnetic field generators necessary for the control of small soft robots comprised of MEs (Manamanchaiyaporn et al., 2020), **(B)** a rigid MRF valve, showing both traditional and jamming designs (Leps et al., 2020), **(C)** a magnetically-selected sME-based joule heater (Yun et al., 2019), and **(D)** an anisotropic hybrid sME with varying resistivity and piezoelectric properties based on the anisotropy angle of the magnetic particles (Yun et al., 2020).

Given the research discussed in this paper, there exist multiple paths forward for further developments. The development of MEs containing both hard and soft powders with different properties will enable more unique electromagnetic applications. One direction would be to create deformable electromagnets, with sMEs providing the core, and flexible windings made of wire or liquid metal providing current to generate a magnetic flux. Such a mechanism would enable the generation of magnetic fields within a soft robot, as opposed to relying upon the application of a distant external magnetic field. This reliance upon an external field is currently one of the largest drawbacks for soft robotics, as while it does succeed in wireless control and actuation, it only does so within highly constrained systems (see Figure 4A). Generating magnetic fields from the soft robot itself opens the path many important robotic actions, such as self-sensing, magnetic coupling with other systems, controlled local deformations, and more.

Electropermanent magnets could also be developed, utilizing both a sME and hME to create the on-off properties of an electromagnet and the persistent properties of a permanent magnet with remanent magnetization. Controllable magnetic fields arising within a soft robot could be used for soft robotic valve control, gating the flow of pressure through a system. This could be accomplished through a physical valve, collapsing or pinching shut, or possibly with the use of MRFs, stopping their flow through a system when exposed to a magnetic field. This was demonstrated recently with hard electropermanent valves and MRFs to create a jamming MRF valve, and could feasibly be expanded upon to allow for fully soft electropermanent magnets made from sMEs and hMEs performing the same action (Leps et al., 2020) (see Figure 4B).

A soft robotic gripper could be made from an elastic electromagnet and a hME, which pinches shut when turned on, grasping objects. Solenoids could be created from flexible wire or liquid metal embedded within elastomers, as this has been shown to be effective for creating flexible circuit elements within elastic substrates (Bira and Mengüç, 2018). These embedded solenoids could measure the state of a deforming soft robotic tentacle as it moves in space, measuring the changing remanent magnetism. These values could be fed into neural nets to better interpret the physical state of the robot (Hellebrekers et al., 2020).

The elastic properties of MEs could also be utilized to generate varying magnetic fields arising from the expansion or compression of the source material. An anisotropic hME with a remanent magnetization could be modulated by its changing shape, allowing for a device where the expansion of the hME directly impacts its magnetic attractive force to other magnetic elements. Most of the research discussed in this review focuses on flexibility or rheological control using external fields, but few attempt to control the electromagnetic properties of the ME through its deformation. Research has shown that the relative positioning and angle of the magnetic particles greatly impacts the resulting magnetic properties of the ME (Hadzir et al., 2019). Some research asserts that the electrical properties of an ME are dependent on both applied mechanical and magnetic changes (Wang et al., 2009), and it can be assumed that the magnetic properties will similarly vary. More recently, researchers created hybrid sMEs containing both carbonyl iron or nickel particles and liquid metal (eutectic Gallium–Indium) (Yun et al., 2019). They demonstrated that the sME displays resistivity changes both from physical deformation as well as an applied magnetic field. Their application allowed for the creation of a selective Joule heater, where positioned magnets locally reduced the resistivity of the sME, enabling selective heating based on the positioning of the magnets (see Figure 4C). In a following publication, they introduced anisotropy to their sMEs through magnetic alignment, and characterized its resistivity and piezoelectric properties in response to changes in strain for different angles of anisotropy (Yun et al., 2020) (see Figure 4D).

## 4. Conclusions

The fundamental properties of MEs are not well-documented, as many disparate areas of research utilize MEs for differing reasons. This review attempts to collect and summarize the state of known properties of MEs, as well as their applications in soft robotics. MEs are currently being used for remote actuation and positioning, damping and vibration control, soft structure deformation, and sensing modalities. Each of these functions has applications within the field of soft robotics, as well as beyond it, and advances in manufacturing techniques, such as 3D printing and programmable magnetic domains during curing of the MEs continues to expand the possibilities of working with these materials. Many studies examine the rheological response of MEs to applied magnetic fields but rely on bulky and highly constrained environments to do so. Little, if any, research exists that attempts to study the magnetic response of MEs in response to dynamic physical changes. While work that analyzes the effects of particle size and concentration on the storage modulus (Böse and Röder, 2009) exists, the authors are unaware of a comprehensive comparison of the magnetic properties of various MEs, with the exception of the magnetic properties of a single hME analyzed by Zhao et al. (2019b). Characterization of various MEs, on both their physical and magnetic properties, will be a valuable starting point for future work on specific applications in soft robotics. This could inform intentional ME composition choices, instead of the occasionally arbitrary choices of a magnetic powder to develop an ME.

The authors anticipate that hMEs will continue to be chosen for remote manipulation applications, such as crawling and swimming soft robots. The use of sMEs in soft robotics will also increase, as their magnetic properties are characterized and differentiated from hMEs to enable alternative sensing and electromagnetic control methods within soft robots. Until better local control mechanisms are developed, the utility of MEs in soft robotics will be limited to scenarios where highly constrained, external magnetic fields can be produced, limiting the potential real-world applications to medical and research settings. Once this significant obstacle is overcome, MEs may become much more common within untethered soft robotic systems, or in more traditional hard robotic systems where sensing and soft interfaces are needed.

## Author Contributions

NB wrote the majority of this article. JD and PD provided the feedback, revisions, and intellectual contributions to the content of the paper through its construction. All authors contributed to the article and approved the submitted version.

## Conflict of Interest

The authors declare that the research was conducted in the absence of any commercial or financial relationships that could be construed as a potential conflict of interest.

## References

[B1] Alfadhel A. Kosel J. (2015). Magnetic nanocomposite cilia tactile sensor. Adv. Mater. 27, 7888–7892. 10.1002/adma.201504015 26487397

[B2] Almansouri A. S. Alsharif N. A. Khan M. A. Swanepoel L. Kaidarova A. Salama K. N. (2019). An imperceptible magnetic skin. Adv. Mater. Technol. 4:1900493 10.1002/admt.201900493

[B3] Behrooz M. Wang X. Gordaninejad F. (2014). Modeling of a new semi-active/passive magnetorheological elastomer isolator. Smart Mater. Struct. 23:045013 10.1088/0964-1726/23/4/045013

[B4] Bellan C. Bossis G. (2002). Field dependence of viscoelastic properties of MR elastomers. Int. J. Mod. Phys. B 16, 2447–2453. 10.1142/S0217979202012499

[B5] Bira N. Mengüç Y. (2018). “Measurement of tissue stiffness using soft EGA-in sensors and pressure application,” in 2018 IEEE International Conference on Soft Robotics (RoboSoft) (Livorno: IEEE), 228–232. 10.1109/ROBOSOFT.2018.8404924

[B6] Böse H. (2007). Viscoelastic properties of silicone-based magnetorheological elastomers. Int. J. Mode. Phys. B 21, 4790–4797. 10.1142/S0217979207045670

[B7] Böse H. Rabindranath R. Ehrlich J. (2012). Soft magnetorheological elastomers as new actuators for valves. J. Intell. Mater. Syst. Struct. 23, 989–994. 10.1177/1045389X11433498

[B8] Böse H. Röder R. (2009). Magnetorheological elastomers with high variability of their mechanical properties. J. Phys. Conf. Series 149:012090 10.1088/1742-6596/149/1/012090

[B9] Bowen L. Springsteen K. Feldstein H. Frecker M. Simpson T. W. von Lockette P. (2015). Development and validation of a dynamic model of magneto-active elastomer actuation of the origami waterbomb base. J. Mech. Robot. 7:011010 10.1115/1.4029290

[B10] Carlson J. D. Jolly M. R. (2000). MR fluid, foam and elastomer devices. Mechatronics 10, 555–569. 10.1016/S0957-4158(99)00064-1

[B11] Chen L. Gong X. Li W. (2007). Microstructures and viscoelastic properties of anisotropic magnetorheological elastomers. Smart Mater. Struct. 16:2645 10.1088/0964-1726/16/6/069

[B12] Ciambella J. Stanier D. C. Rahatekar S. S. (2017). Magnetic alignment of short carbon fibres in curing composites. Compos. B Eng. 109, 129–137. 10.1016/j.compositesb.2016.10.038

[B13] Coquelle E. Bossis G. (2006). Mullins effect in elastomers filled with particles aligned by a magnetic field. Int. J. Solids Struct. 43, 7659–7672. 10.1016/j.ijsolstr.2006.03.020

[B14] Damiani R. Sun L. (2017). Microstructural characterization and effective viscoelastic behavior of magnetorheological elastomers with varying acetone contents. Int. J. Damage Mech. 26, 104–118. 10.1177/1056789516657676

[B15] Davis L. (1999). Model of magnetorheological elastomers. J. Appl. Phys. 85, 3348–3351. 10.1063/1.369682

[B16] Dorfmann A. Ogden R. (2003). Magnetoelastic modelling of elastomers. Eur. J. Mech. A Solids 22, 497–507. 10.1016/S0997-7538(03)00067-6

[B17] Gong X. Zhang X. Zhang P. (2005). Fabrication and characterization of isotropic magnetorheological elastomers. Polym. Test. 24, 669–676. 10.1016/j.polymertesting.2005.03.015

[B18] Gordaninejad F. Breese D. G. (1999). Heating of magnetorheological fluid dampers. J. Intell. Mater. Syst. Struct. 10, 634–645. 10.1106/55D1-XAXP-YFH6-B2FB

[B19] Gorissen B. Reynaerts D. Konishi S. Yoshida K. Kim J.-W. De Volder M. (2017). Elastic inflatable actuators for soft robotic applications. Adv. Mater. 29:1604977. 10.1002/adma.201604977 28949425

[B20] Gudmundsson I. (2011). A feasibility study of magnetorheological elastomers for a potential application in prosthetic devices (Ph.D. thesis), University of Iceland, Reykjavík, Iceland.

[B21] Hadzir M. N. H. Bakar M. H. A. Azid I. A. (2019). “Effect of the magnetic field on magnetic particles in magnetorheological elastomer layers,” in Advanced Engineering for Processes and Technologies eds A. Ismail, M. H. Abu Bakar, and A. Öchsner (New York, NY: Springer), 135–143. 10.1007/978-3-030-05621-6_11

[B22] Hellebrekers T. Chang N. Chin K. Ford M. Kroemer O. Majidi C. (2020). Soft magnetic tactile skin for continuous force and location estimation using neural networks. IEEE Robot. Autom. Lett. 5, 3892–3898. 10.1109/LRA.2020.2983707

[B23] Hilber W. (2016). Stimulus-active polymer actuators for next-generation microfluidic devices. Appl. Phys. A 122:751 10.1007/s00339-016-0258-6

[B24] Hu W. Lum G. Z. Mastrangeli M. Sitti M. (2018). Small-scale soft-bodied robot with multimodal locomotion. Nature 554, 81–85. 10.1038/nature25443 29364873

[B25] Hua D. Liu X. Sun S. Sotelo M. A. Li Z. Li W. (2020). A magnetorheological fluid filled soft crawling robot with magnetic actuation. IEEE/ASME Trans. Mechatron. 10.1109/TMECH.2020.2988049

[B26] Jeon S. Hoshiar A. K. Kim K. Lee S. Kim E. Lee S.. (2019). A magnetically controlled soft microrobot steering a guidewire in a three-dimensional phantom vascular network. Soft Robot. 6, 54–68. 10.1089/soro.2018.0019 30312145 PMC6386781

[B27] Jolly M. R. Bender J. W. Carlson J. D. (1999). Properties and applications of commercial magnetorheological fluids. J. Intell. Mater. Syst. Struct. 10, 5–13. 10.1177/1045389X9901000102

[B28] Jolly M. R. Carlson J. D. Munoz B. C. (1996a). A model of the behaviour of magnetorheological materials. Smart Mater. Struct. 5:607 10.1088/0964-1726/5/5/009

[B29] Jolly M. R. Carlson J. D. Muñoz B. C. Bullions T. A. (1996b). The magnetoviscoelastic response of elastomer composites consisting of ferrous particles embedded in a polymer matrix. J. Intell. Mater. Syst. Struct. 7, 613–622. 10.1177/1045389X9600700601

[B30] Joyee E. B. Pan Y. (2019). A fully three-dimensional printed inchworm-inspired soft robot with magnetic actuation. Soft Robot. 6, 333–345. 10.1089/soro.2018.0082 30720388

[B31] Ju B. Yu M. Fu J. Yang Q. Liu X. Zheng X. (2012). A novel porous magnetorheological elastomer: preparation and evaluation. Smart Mater. Struct. 21:035001 10.1088/0964-1726/21/3/035001

[B32] Jung H. S. Kwon S. H. Choi H. J. Jung J. H. Kim Y. G. (2016). Magnetic carbonyl iron/natural rubber composite elastomer and its magnetorheology. Compos. Struct. 136, 106–112. 10.1016/j.compstruct.2015.10.008

[B33] Kallio M. (2005). The Elastic and Damping Properties of Magnetorheological Elastomers. Finland: VTT Espoo.

[B34] Kashima S. Miyasaka F. Hirata K. (2012). Novel soft actuator using magnetorheological elastomer. IEEE Trans. Magn. 48, 1649–1652. 10.1109/TMAG.2011.2173669

[B35] Kavlicoglu B. Gordaninejad F. Evrensel C. Fuchs A. Korol G. (2006). A semi-active, high-torque, magnetorheological fluid limited slip differential clutch. J. Vib. Acoust. 128, 604–610. 10.1115/1.2203308

[B36] Kim J. Chung S. E. Choi S.-E. Lee H. Kim J. Kwon S. (2011). Programming magnetic anisotropy in polymeric microactuators. Nat. Mater. 10, 747–752. 10.1038/nmat3090 21822261

[B37] Kim Y. Parada G. A. Liu S. Zhao X. (2019). Ferromagnetic soft continuum robots. Sci. Robot. 4:eaax7329 10.1126/scirobotics.aax7329 33137788

[B38] Kim Y. Yuk H. Zhao R. Chester S. A. Zhao X. (2018). Printing ferromagnetic domains for untethered fast-transforming soft materials. Nature 558:274. 10.1038/s41586-018-0185-0 29899476

[B39] Kitano S. Komatsuzaki T. Suzuki I. Nogawa M. Naito H. Tanaka S. (2020). Development of a rigidity tunable flexible joint using magneto-rheological compounds—toward a multijoint manipulator for laparoscopic surgery. Front. Robot. AI 7:59 10.3389/frobt.2020.00059 PMC7805682 33501227

[B40] Lee C. Kim M. Kim Y. J. Hong N. Ryu S. Kim H. J. (2017). Soft robot review. Int. J. Control Autom. Syst. 15, 3–15. 10.1007/s12555-016-0462-3

[B41] Leng D. Wu T. Liu G. Wang X. Sun L. (2018). Tunable isolator based on magnetorheological elastomer in coupling shear-squeeze mixed mode. J. Intell. Mater. Syst. Struct. 29, 2236–2248. 10.1177/1045389X18758205

[B42] Leps T. J. Glick P. Ruffatto I. I. I. D Parness A. Tolley M. T. (2020). A low-power, jamming, magnetorheological valve using electropermanent magnets suitable for distributed control in soft robots. Smart Mater. Struct. 29:105025 10.1088/1361-665X/abadd4

[B43] Li Y. Li J. Li W. Du H. (2014). A state-of-the-art review on magnetorheological elastomer devices. Smart Mater. Struct. 23:123001 10.1088/0964-1726/23/12/123001

[B44] Lokander M. Stenberg B. (2003). Performance of isotropic magnetorheological rubber materials. Polym. Test. 22, 245–251. 10.1016/S0142-9418(02)00043-0

[B45] Lu N. Kim D.-H. (2014). Flexible and stretchable electronics paving the way for soft robotics. Soft Robot. 1, 53–62. 10.1089/soro.2013.0005

[B46] Lum G. Z. Ye Z. Dong X. Marvi H. Erin O. Hu W.. (2016). Shape-programmable magnetic soft matter. Proc. Natl. Acad. Sci. U.S.A. 113, E6007–E6015. 10.1073/pnas.1608193113 27671658 PMC5068264

[B47] Manamanchaiyaporn L. Xu T. Wu X. (2020). Magnetic soft robot with the triangular head-tail morphology inspired by lateral undulation. IEEE/ASME Trans. Mechatron. 10.1109/TMECH.2020.2988718

[B48] Nayak B. Dwivedy S. K. Murthy K. S. (2015). Fabrication and characterization of magnetorheological elastomer with carbon black. J. Intell. Mater. Syst. Struct. 26, 830–839. 10.1177/1045389X14535011

[B49] Pacchierotti C. Ongaro F. Van den Brink F. Yoon C. Prattichizzo D. Gracias D. H.. (2017). Steering and control of miniaturized untethered soft magnetic grippers with haptic assistance. IEEE Trans. Autom. Sci. Eng. 15, 290–306. 10.1109/TASE.2016.2635106 31423113 PMC6697175

[B50] Qi S. Guo H. Fu J. Xie Y. Zhu M. Yu M. (2020). 3d printed shape-programmable magneto-active soft matter for biomimetic applications. Compos. Sci. Technol. 188:107973 10.1016/j.compscitech.2019.107973

[B51] Rus D. Tolley M. T. (2015). Design, fabrication and control of soft robots. Nature 521, 467–475. 10.1038/nature14543 26017446

[B52] Rus D. Tolley M. T. (2018). Design, fabrication and control of origami robots. Nat. Rev. Mater. 3:101. 10.1038/s41578-018-0009-8 26017446

[B53] Sung C. Lin R. Miyashita S. Yim S. Kim S. Rus D. (2017). “Self-folded soft robotic structures with controllable joints,” in 2017 IEEE International Conference on Robotics and Automation (ICRA) (Singapore: IEEE), 580–587. 10.1109/ICRA.2017.7989072

[B54] Tang J. Tong Z. Xia Y. Liu M. Lv Z. Gao Y.. (2018). Super tough magnetic hydrogels for remotely triggered shape morphing. J. Mater. Chem. B 6, 2713–2722. 10.1039/C8TB00568K 32254223

[B55] Wan Y. Xiong Y. Zhang S. (2018). Temperature effect on viscoelastic properties of anisotropic magnetorheological elastomers under compression. Smart Mater. Struct. 28:015005 10.1088/1361-665X/aaeaf8

[B56] Wang X. Gordaninejad F. Calgar M. Liu Y. Sutrisno J. Fuchs A. (2009). Sensing behavior of magnetorheological elastomers. J. Mech. Des. 131:091004 10.1115/1.3160316

[B57] Whitesides G. M. (2018). Soft robotics. Angew. Chem. Int. Ed. 57, 4258–4273. 10.1002/anie.201800907 29517838

[B58] Wu C. Fan X. Zhang Q. Wang W. Song Y. Zheng Q. (2020). Magnetorheological elastomer peristaltic pump capable of flow and viscosity control. J. Intell. Mater. Syst. Struct. 31:1045389X20916803 10.1177/1045389X20916803

[B59] Wu C. Zhang Q. Fan X. Song Y. Zheng Q. (2019). Smart magnetorheological elastomer peristaltic pump. J. Intell. Mater. Syst. Struct. 30, 1084–1093. 10.1177/1045389X19828825

[B60] Yun G. Tang S.-Y. Sun S. Yuan D. Zhao Q. Deng L.. (2019). Liquid metal-filled magnetorheological elastomer with positive piezoconductivity. Nat. Commun. 10, 1–9. 10.1038/s41467-019-09325-4 30899009 PMC6428896

[B61] Yun G. Tang S.-Y. Zhao Q. Zhang Y. Lu H. Yuan D. (2020). Liquid metal composites with anisotropic and unconventional piezoconductivity. Matter 3, 824–841. 10.1016/j.matt.2020.05.022

[B62] Zajac P. Kaleta J. Lewandowski D. Gasperowicz A. (2010). Isotropic magnetorheological elastomers with thermoplastic matrices: structure, damping properties and testing. Smart Mater. Struct. 19:045014 10.1088/0964-1726/19/4/045014

[B63] Zhang J. Diller E. (2018). Untethered miniature soft robots: Modeling and design of a millimeter-scale swimming magnetic sheet. Soft Robot. 5, 761–776. 10.1089/soro.2017.0126 30256177

[B64] Zhang L. Abbott J. J. Dong L. Kratochvil B. E. Bell D. Nelson B. J. (2009a). Artificial bacterial flagella: fabrication and magnetic control. Appl. Phys. Lett. 94:064107 10.1063/1.3079655

[B65] Zhang W. Gong X. Li J. Zhu H. Jiang W. (2009b). Radiation vulcanization of magnetorheological elastomers based on silicone rubber. Chin. J. Chem. Phys. 22, 535–540. 10.1088/1674-0068/22/05/535-540

[B66] Zhao D. Wang B. Zhao Z. Wang S. Yang S. Dong N. (2019a). The simulation of magneto-mechanical properties of magnetorheological elastomers. IOP Conf. Series Mater. Sci. Eng. 649:012003 10.1088/1757-899X/649/1/012003

[B67] Zhao R. Kim Y. Chester S. A. Sharma P. Zhao X. (2019b). Mechanics of hard-magnetic soft materials. J. Mech. Phys. Solids 124, 244–263. 10.1016/j.jmps.2018.10.008

[B68] Zhou G. (2003). Shear properties of a magnetorheological elastomer. Smart Mater. Struct. 12:139 10.1088/0964-1726/12/1/316

